# Novel dyssynchrony burden index and right ventricular function for mortality risk stratification in right ventricular apical pacing – a retrospective cohort study

**DOI:** 10.1016/j.ijcha.2026.102019

**Published:** 2026-09-23

**Authors:** Marwin Bannehr, Laura David, Christoph Edlinger, Christian Georgi, Paulina Karlsson, Tanja Kücken, Martin Seifert, Anja Haase-Fielitz, Christian Butter

**Affiliations:** a Department of Cardiology, Heart Center Brandenburg, Brandenburg Medical School (MHB) Theodor Fontane, Bernau/Berlin, Germany; b Faculty of Health Sciences (FGW), Joint Faculty of the University of Potsdam, the Brandenburg Medical School Theodor Fontane and the Brandenburg Technical University Cottbus-Senftenberg, Cottbus, Germany; c Institute of Social Medicine and Health System Research, Otto von Guericke University Magdeburg, Magdeburg, Germany

**Keywords:** Right ventricular pacing, QRS duration, Pacing burden, Right ventricular function, TAPSE, RV strain, Pacemaker induced cardiomyopathy, Survival, Mortality

## Abstract

**Background:**

Right ventricular (RV) apical pacing induces electrical and mechanical dyssynchrony, potentially causing adverse remodelling and mortality. The combined prognostic effect of paced QRS duration, RV pacing burden, and RV dysfunction remains insufficiently characterized.

**Methods:**

This retrospective cohort study analyzed 180 patients with permanent pacemakers, RV apical pacing, and LVEF ≥40%. RV function was assessed by TAPSE and RV free-wall strain. A dyssynchrony burden index (standardized sum of QRS duration and pacing burden) was developed, alongside a simple 0–2 point score using clinical (QRS >150 ms, burden >40%) and Youden-optimal (>159 ms, >43%) thresholds. ROC, Kaplan-Meier, and Cox regression analyses assessed two-year all-cause mortality.

**Results:**

Twenty patients (11.1%) died. Non-survivors showed higher pacing burden, QRS duration, and impaired RV function (all *p* < 0.001). The continuous index showed superior discrimination (AUC 0.832) and was an independent Cox predictor (HR 3.886, *p* < 0.001), as were TAPSE and LVEF. Combining the index with TAPSE or RV strain raised AUC to 0.917 and 0.981. Both point-scores were independently predictive (OR per point 5.17 and 5.31, *p* < 0.001) with only modestly lower discrimination (AUC 0.777, clinical thresholds, *p* = 0.005; AUC 0.796, Youden thresholds, *p* = 0.063, *p* = 0.063) vs. continuous index. A point score ≤ 1 was highly sensitive (95%, NPV ≥98%) for rule-out, and a score of 2 reasonably specific (71–77%, LR+ 2.8–3.2) for rule-in.

**Conclusions:**

The dyssynchrony burden index is an independent predictor of mortality in RV apical pacing; RV dysfunction adds complementary value. A simplified, bedside-calculable point-based version preserves most of this prognostic information, offering a practical risk-stratification tool.

## Graphical abstract

**Figure:**
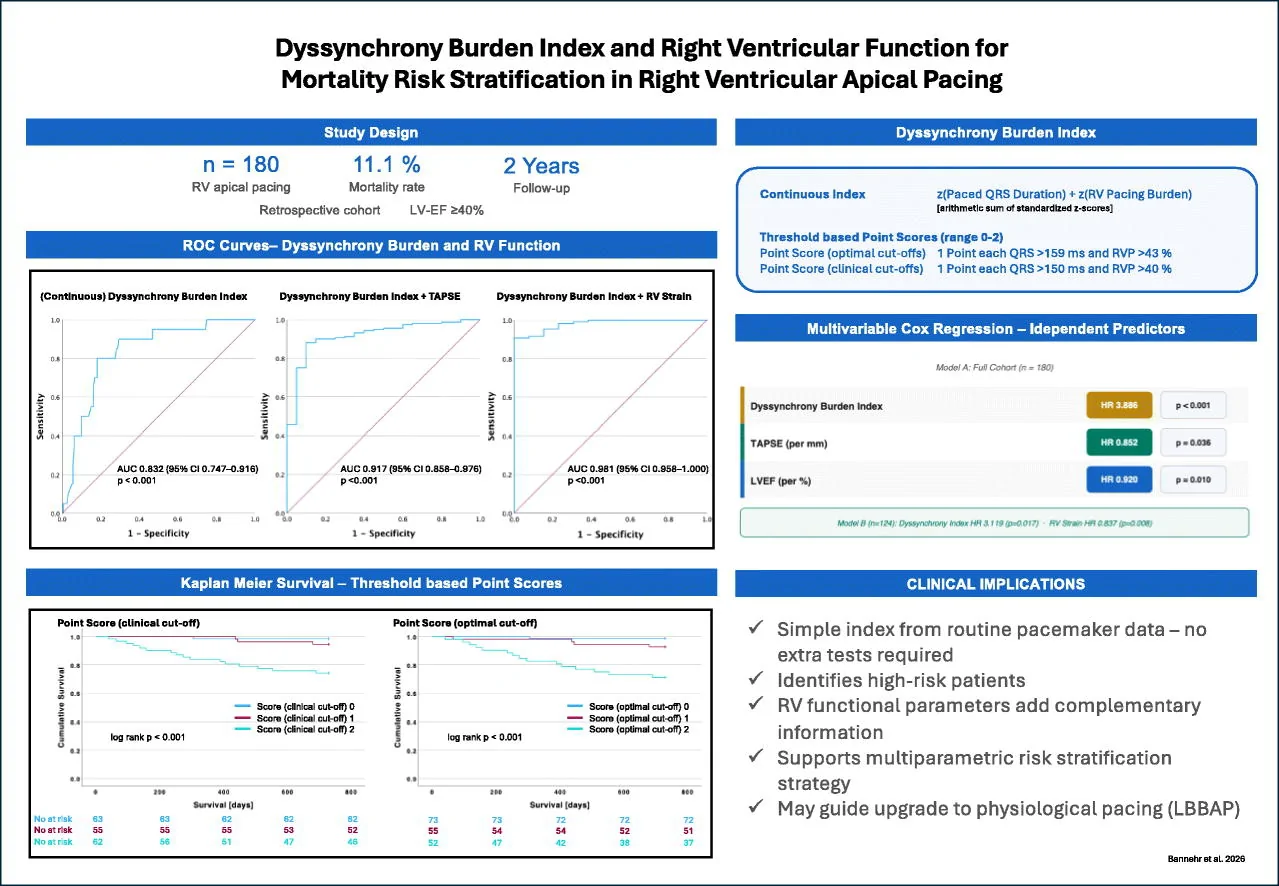

## 1 Background

Right ventricular (RV) apical pacing has been the dominant mode of ventricular stimulation since the introduction of permanent cardiac pacing more than six decades ago. While it reliably restores an adequate ventricular rate, its electromechanical consequences have become a subject of sustained investigation [1]. Stimulation at the RV apex generates an abnormal sequence of ventricular activation, with the electrical wavefront propagating from right to left and from apex to base rather than following the His-Purkinje axis. The resulting interventricular and intraventricular dyssynchrony produces heterogeneous myocardial shortening, regional wall motion abnormalities, abnormal septal motion, and paradoxical interventricular septal shift – collectively initiating a cascade of adverse structural and functional remodelling [2].

Epidemiological evidence of harm from RV apical pacing emerged prominently from large, randomised trials. The Dual Chamber and VVI Implantable Defibrillator (DAVID) trial demonstrated that programming ICDs to DDDR 70 ppm, which imposed high RV pacing rates, significantly increased the composite of death and heart failure hospitalisation compared with VVI 40 backup pacing, establishing that unnecessary RV stimulation is not innocuous [3]. Subsequently, the Mode Selection Trial (MOST) revealed that a higher percentage of ventricular pacing was independently associated with an increased risk of heart failure hospitalisation and atrial fibrillation in patients with sick sinus syndrome, with each percentage-point increase in cumulative ventricular pacing conferring a 2–3% incremental risk [4]. The DANPACE trial and the long-term SAVE PACe data further reinforced the deleterious effects of high-burden RV pacing in pacemaker populations [5], [6].

The pathophysiology underlying pacing-induced cardiomyopathy (PICM) is multifactorial [7]. At the cellular level, heterogeneous mechanical load imposed by dyssynchronous activation leads to differential myocyte hypertrophy, fibrosis, and altered calcium handling in early-activated versus late-activated segments [8]. At the organ level, these changes manifest as progressive LV dilatation, mitral regurgitation, and a decline in LV ejection fraction (LVEF). PICM is conventionally defined as a drop in LVEF of 10 percentage points or more to below 50% in the setting of high-burden RV pacing, occurring in approximately 10–20% of patients [9]. Risk factors include pacing burden above 20–40%, pre-existing LV impairment, male sex, and broader paced QRS duration [10]. The paced QRS duration has gained attention as an independently actionable marker: it reflects both the site of stimulation (with apical locations generating wider complexes than physiological pacing alternatives) and inter-individual differences in ventricular activation reserve.

Beyond LV dysfunction, RV apical pacing exerts direct and indirect effects on RV function itself. The mechanical interaction between the ventricles – ventricular interdependence – means that dyssynchronous LV contraction imposes abnormal loading conditions on the RV through shared myocardial fibres and the interventricular septum [11]. Chronic RV apical pacing may therefore lead to RV remodelling manifest as dilatation and longitudinal dysfunction, as quantified by tricuspid annular plane systolic excursion (TAPSE) and speckle-tracking-derived RV free-wall longitudinal strain. These parameters have emerged as robust prognostic markers across diverse cardiac conditions, yet their specific prognostic role in the context of pacing-induced dyssynchrony has been examined only in small, heterogeneous cohorts [12], [13].

A critical knowledge gap persists regarding the combined assessment of the electrical dyssynchrony stimulus – quantified jointly by paced QRS duration and RV pacing burden – and the resultant RV functional impairment as dual, potentially complementary determinants of long-term survival. Existing prognostic models have assessed pacing burden and QRS duration in isolation or have focused primarily on LV remodelling outcomes, without integrating a composite electrical exposure metric with a structured evaluation of both conventional and advanced RV function parameters. Furthermore, most prior studies have either included patients with pre-existing LV systolic dysfunction – thereby confounding outcomes – or have lacked adequate echocardiographic phenotyping of the right heart.

The present study aims to develop a simple, clinically applicable risk score that integrates electrical exposure (the dyssynchrony index) with right ventricular function (TAPSE or RV free-wall longitudinal strain) to improve mortality prediction in patients with right ventricular apical pacing and preserved left ventricular ejection fraction. The central hypothesis is that cumulative electrical dyssynchrony exposure and RV dysfunction represent pathophysiological substrates, whose integration yields superior risk stratification compared with either domain assessed in isolation.

## 2 Methods

### 2.1 Study design and population

In this single-center retrospective observational cohort study, data from an institutional echo database consisting of 1960 consecutive patients undergoing echocardiography a tertiary care centre in Brandenburg, Germany were screened. 310 were lost follow-up. Of the remaining 1650 patients 396 had a cardiac implantable electronic device. Patients with defibrillators, septal or biventricular pacing devices, and LV systolic dysfunction (LVEF <40%), were excluded. Finally, the study population comprised 180 patients with RV apical pacing. The patient flow is shown in Fig. 1.

**Fig. 1:**
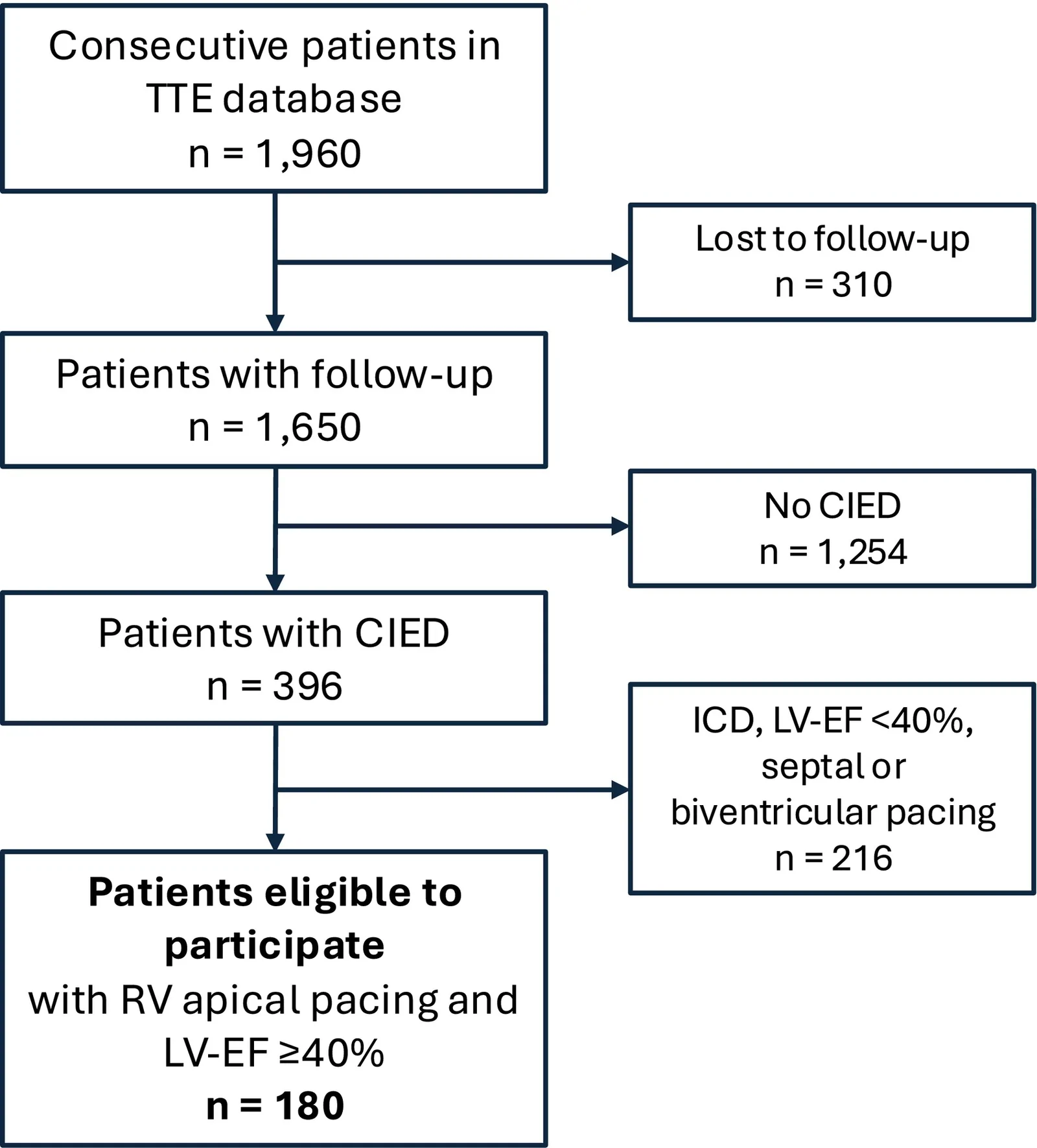
**Strobe diagram of the patient flow.** Figure shows patients included and excluded in the study with a number of “n” individuals at each step. TTE = transthoracic echocardiography, CIED = cardiac implantable electronic device, ICD = implantable cardioverter defibrillator, LV-EF = left ventricular ejection fraction, RV = right ventricular.

### 2.2 Data collection

Clinical, ECG, device, and echocardiographic data were collected retrospectively from medical records and device clinic reports. Baseline demographics were recorded. Device interrogation reports provided the cumulative RV pacing burden (%), defined as the percentage of ventricular beats that were paced. Data for survival rates was collected from hospital database and primary care physicians.

“Time zero” was defined as the date of the index pacemaker interrogation and qualifying echocardiographic assessment, which were performed concurrently; patients were followed from this date for two-year all-cause mortality. The median time from pacemaker implantation to this index assessment was 1100 days, reflecting that paced QRS duration and RV pacing burden were measured after a period of chronic pacing exposure had already occurred. This design reflects the clinical reality that patients typically present for risk assessment at a routine device follow-up visit.

### 2.3 Ethics

The study was approved by the local Ethics Committee of the State Brandenburg (AS 155(bB)/2017) and was conducted in accordance with the 1975 Declaration of Helsinki. Informed consent was not necessary to obtain according to the ethic committee's statement because of the retrospective nature of this study. The Strengthening the Reporting of Observational Studies in Epidemiology recommendations for reporting observational studies were applied.

### 2.4 Electrocardiographic assessment

The paced QRS duration (ms) was measured from surface 12‑lead electrocardiograms obtained during pacemaker follow-up visits, reflecting activation time during RV apical pacing. QRS duration was measured by two independent investigators from 12‑lead ECGs. Discrepancies were resolved by consensus.

Only clearly fully-paced QRS complexes were measured; complexes with visible fusion or pseudofusion were excluded from QRS duration measurement. RV pacing burden, was taken directly from the device-reported cumulative percentage of paced ventricular beats, which is not able to distinguish true ventricular capture from fusion or pseudofusion beats.

### 2.5 Echocardiographic assessment

Standard transthoracic echocardiography was performed according to current guidelines. Left ventricular ejection fraction was assessed using the biplane Simpson method. RV size was quantified using basal (RVD1), mid-cavity (RVD2), and longitudinal (RVD3) dimensions. RV function was evaluated by two complementary parameters: 1) Tricuspid annular plane systolic excursion (TAPSE, mm), measured by M-mode echocardiography from the apical four-chamber view; 2) RV free-wall longitudinal strain (%), obtained by two-dimensional speckle-tracking echocardiography (available in a subset of *n* = 124 patients). TAPSE and RV free-wall longitudinal strain were measured by three cardiologists with experience in cardiac imaging.

### 2.6 Dyssynchrony burden index

Three composite metrics were evaluated to capture the cumulative electrical exposure to RV apical pacing dyssynchrony: 1) To create a composite measure of paced QRS duration and RV pacing burden, the two component variables were standardized by converting each variable to a z-score. The standardized scores were then summed to generate a composite index (Dyssynchrony Burden Index = [zPaced QRS Duration] + z[RV Pacing Burden]), with higher values indicating greater levels of the dyssynchrony index, 2) the product of both variables, which models a multiplicative interaction, and 3) a statistical interaction term which accounts for synergistic effects between QRS duration and pacing burden (QRS duration + RV pacing burden + [QRS duration * RV pacing burden]).

The arithmetic sum of the standardized z-scores was selected as the primary index due to its superior predictive performance and a point-based scoring system based on two sets of thresholds was developed as a practical companion tool.

### 2.7 Statistical analysis

Data were assessed for normality using histograms. Continuous variables are reported as mean ± standard deviation or median with interquartile range (IQR) as appropriate. Group comparisons between survivors and non-survivors were performed using independent-samples *t*-tests.

ROC curve analyses with calculation of areas under the curve (AUC) were performed for each predictor variable and composite index with respect to two-year all-cause mortality. AUC comparisons were performed using the method of Hanley and McNeil.

As a sensitivity analysis addressing the clinical applicability of the continuous z-score index, a simple integer point-based dyssynchrony burden score was additionally constructed by dichotomising paced QRS duration and RV pacing burden at two sets of pre-specified thresholds: 1) round clinical thresholds, and 2) the Youden-optimal cut-offs derived from this cohorts' ROC analyses; one point was assigned for each criterion met. Discrimination of both point-based scores was compared with the continuous z-score index using DeLong non-parametric method for correlated ROC curves. Associations with two-year mortality were assessed by univariable and age-adjusted logistic regression, and clinical performance (sensitivity, specificity, positive and negative predictive value, likelihood ratios) was calculated at score thresholds.

The dyssynchrony burden index was stratified into quartiles, and patients above the 75th percentile were dichotomized for Kaplan-Meier survival analysis; group differences were evaluated with the log-rank test.

Multivariable Cox proportional hazards regression was performed in two models: Model A included age, sex, TAPSE, LVEF, and dyssynchrony burden index; Model B replaced TAPSE with RV free-wall strain.

Finally, ROC analyses were performed for combined models incorporating both the dyssynchrony index and RV functional parameters (TAPSE or RV strain) using the predicted probabilities from logistic regression.

Internal validation of the multivariable Cox models was performed using bootstrap resampling (1000 resamples with replacement), reporting the apparent C-statistic, the optimism-corrected C-statistic, and the bootstrap-derived shrinkage factor for each model, in view of the limited number of events relative to the number of covariates.

An AUC ≥0.700 was considered acceptable, ≥0.800 good, and ≥ 0.90 excellent. Statistical significance was defined as *p* < 0.05. All analyses were performed using SPSS Version 29.0 (IBM Corporation).

## 3 Results

### 3.1 Study population and events

The study included 180 patients (mean age 74.6 ± 11.4 years; 56.1% male) who had permanent pacemakers with a RV apical pacing lead and a left ventricular ejection fraction ≥40% at the time of echocardiographic assessment. A total of 20 patients (11.1%) died during the two-year observation period. Right ventricular free-wall strain data were available in a subset of 124 patients (15 deceased, 109 alive). Baseline clinical, echocardiographic, and electrophysiological characteristics of the overall cohort and comparisons between survivors and non-survivors are presented in Table 1.

**Table 1:** Clinical, Echocardiographic, and Electrophysiological Parameters Overall and Separated by Survival Status at Two-Year Follow-Up.

**Variable**	**Overall *n* = 180**	**Survivors *n* = 160**	**Non-Survivors *n* = 20**	**p**
**Demographics**				
Age [years]	74.6 ± 11.4	74.0 ± 11.9	79.0 ± 6.4	0.048
Sex male (%)	101 (56.1)	89 (55.6)	15 (75.0)	0.377
BMI [kg/m^2^]	28.1 ± 4.6	28.2 ± 4.8	27.3 ± 2.9	0.441
**Pacemaker Programming**				0.062
DDD/DDDR (%)	99 (55.0)	96 (60.0)	3 (15.0)	
DDI/DDIR (%)	18 (10.0)	15 (9.4)	3 (15.0)	
VVI/VVIR (%)	63 (35.0)	55 (34.4)	14 (70.0)	
**ECG and Device Parameters**				
Heart rate [/min]	74.2 ± 18.5	74.7 ± 15.4	70.5 ± 18.9	0.352
Paced QRS duration [ms]	138.6 ± 35.8	134.9 ± 35.1	166.0 ± 28.6	<0.001
Intrinsic QRS duration [ms] *	129.6 ± 32.5	128.9 ± 33.3	138.3 ± 17.4	0.495
RV pacing burden [%]	55.7 ± 39.5	51.6 ± 39.3	88.7 ± 23.6	<0.001
RA pacing burden [%]	33.1 ± 35.2	32.4 ± 35.5	52.0 ± 22.3	0.346
AV interval [ms]	195.0 ± 55.8	194.7 ± 55.9	198.7 ± 59.4	0.869
**Echocardiography – Left Ventricle**				
LVEDD [mm]	49.4 ± 7.7	49.2 ± 7.8	50.5 ± 6.3	0.460
LVESD [mm]	36.9 ± 8.5	36.8 ± 8.7	38.0 ± 7.1	0.505
LV-EF [%]	52.4 ± 8.4	53.4 ± 7.9	45.1 ± 8.1	<0.001
**Echocardiography – Right Ventricle**				
RVD1 – Basal diameter [mm]	36.8 ± 7.2	36.3 ± 7.0	40.6 ± 7.1	0.015
RVD2 – Mid-cavity diameter [mm]	27.7 ± 6.8	27.3 ± 6.9	30.4 ± 5.3	0.065
RVD3 – Longitudinal diameter [mm]	65.6 ± 10.0	64.8 ± 9.8	70.6 ± 10.5	0.020
TAPSE [mm]	20.5 ± 4.4	21.0 ± 4.4	16.4 ± 2.2	<0.001
RV free-wall strain† [%]	21.2 ± 7.5	22.4 ± 6.9	12.2 ± 4.9	<0.001
RA dilation ≥ moderate (%)	25 (13.9)	25 (15.6)	4 (20.0)	0.494
LA dilation ≥ moderate (%)	51 (28.3)	46 (29.4)	5 (25.0)	0.857
**Laboratory and Functional Parameters**				
NT-proBNP [ng/l]	3169 ± 4058	2907 ± 3882	5265 ± 4958	0.057
CKD-EPI GFR [ml/min]	61.6 ± 27.1	64.3 ± 27.4	43.4 ± 16.1	0.002
**Comorbidities**				
Coronary artery disease (%)	107 (59.4)	93 (58.1)	14 (70.0)	0.614
Hypertension (%)	177 (98.3)	157 (98.1)	20 (100.0)	0.356
Dyslipidaemia (%)	128 (71.1)	111 (69.4)	17 (85.0)	0.467
Atrial fibrillation (%)	96 (53.3)	84 (52.5)	12 (60.0)	0.637
AF subtype – Paroxysmal (%)	42 (23.3)	37 (23.1)	5 (25.0)	
AF subtype – Persistent (%)	20 (11.1)	18 (11.3)	2 (10.0)	
AF subtype – Permanent (%)	34 (18.9)	29 (18.1)	5 (25.0)	
Type 2 diabetes mellitus (%)	68 (37.8)	56 (35.0)	12 (60.0)	0.097
COPD (%)	30 (16.7)	27 (16.9)	3 (15.0)	1.000
**Guideline-Directed Medical Therapy**				
ACE inhibitor / ARB / ARNI (%)	138 (76.7)			
Beta-blocker (%)	96 (53.3)			
Torasemide (%)	97 (53.9)			
Aldosterone antagonist (%)	40 (22.2)			
Hydrochlorothiazide (%)	30 (16.7)			
SGLT2 inhibitor (%)	21 (11.7)			
Furosemide (%)	7 (3.9)			
Xipamide (%)	5 (2.8)			

Data are mean ± standard deviation or n (%). * In case of (intermittent) intrinsic atrioventricular conduction. † Subset of *n* = 124 patients.

Abbreviations: BMI = body mass index; RV = right ventricle; RA = right atrium; AV = atrioventricular; LVEDD = left ventricular end-diastolic diameter; LVESD = left ventricular end-systolic diameter; LV-EF = left ventricular ejection fraction; RVD = right ventricular diameter; TAPSE = tricuspid annular plane systolic excursion; NT-proBNP = N-terminal pro-brain natriuretic peptide; GFR = glomerular filtration rate; AF = atrial fibrillation; COPD = chronic obstructive pulmonary disease; LA = left atrium.

### 3.2 Comparison of survivors and non-survivors

Non-survivors were older (79.0 ± 6.4 vs. 74.0 ± 11.9 years, *p* = 0.048) and more often had VVI/VVIR pacemaker programming (70.0% vs. 30.6%), whereas DDD/DDDR programming was more common among survivors (60.0% vs. 15.0%; overall *p* = 0.062 for programming mode). Non-survivors demonstrated significantly higher RV pacing burden (88.7 ± 23.6% vs. 51.6 ± 39.3%), broader paced QRS duration (166.0 ± 28.6 ms vs. 134.9 ± 35.1 ms), reduced TAPSE (16.4 ± 2.2 mm vs. 21.0 ± 4.4 mm), markedly impaired RV free-wall strain (12.2 ± 4.9% vs. 22.4 ± 6.9%), lower LV-EF (45.1 ± 8.1% vs. 53.4 ± 7.9%), dilated right ventricular dimensions (RVD1 40.6 ± 7.1 mm vs. 36.3 ± 7.0 mm; RVD3 70.6 ± 10.5 mm vs. 64.8 ± 9.8 mm), and impaired renal function (CKD-EPI GFR 43.4 ± 16.1 vs. 64.3 ± 27.4 ml/min/1.73m^2^) compared to survivors (all *p* ≤ 0.020). NT-proBNP was numerically higher in non-survivors (5265 ± 4958 vs. 2907 ± 3882 ng/l, *p* = 0.057). Comorbidity burden (coronary artery disease, hypertension, atrial fibrillation, type 2 diabetes mellitus, COPD) and LV dimensions did not differ significantly between groups.

### 3.3 Dyssynchrony burden index: construction and distribution

Among three tested composite metrics – the arithmetic sum of the standardized z-scores, the product, and the statistical interaction term of paced QRS duration and RV pacing burden – the arithmetic sum yielded the highest AUC for two-year mortality prediction (AUC 0.832, 95% CI 0.747–0.916) and was therefore designated the primary dyssynchrony burden index (Fig. 2). The median dyssynchrony burden index in the cohort was −0.631 (25th percentile: −1.453; 75th percentile: 1.644). In the quartile analysis, the highest-risk stratum (index >1.644; 4th quartile, *n* = 45) accounted for 16 of 20 deaths (80%), compared to ≤2 deaths in each of the three lower quartiles.

**Fig. 2:**
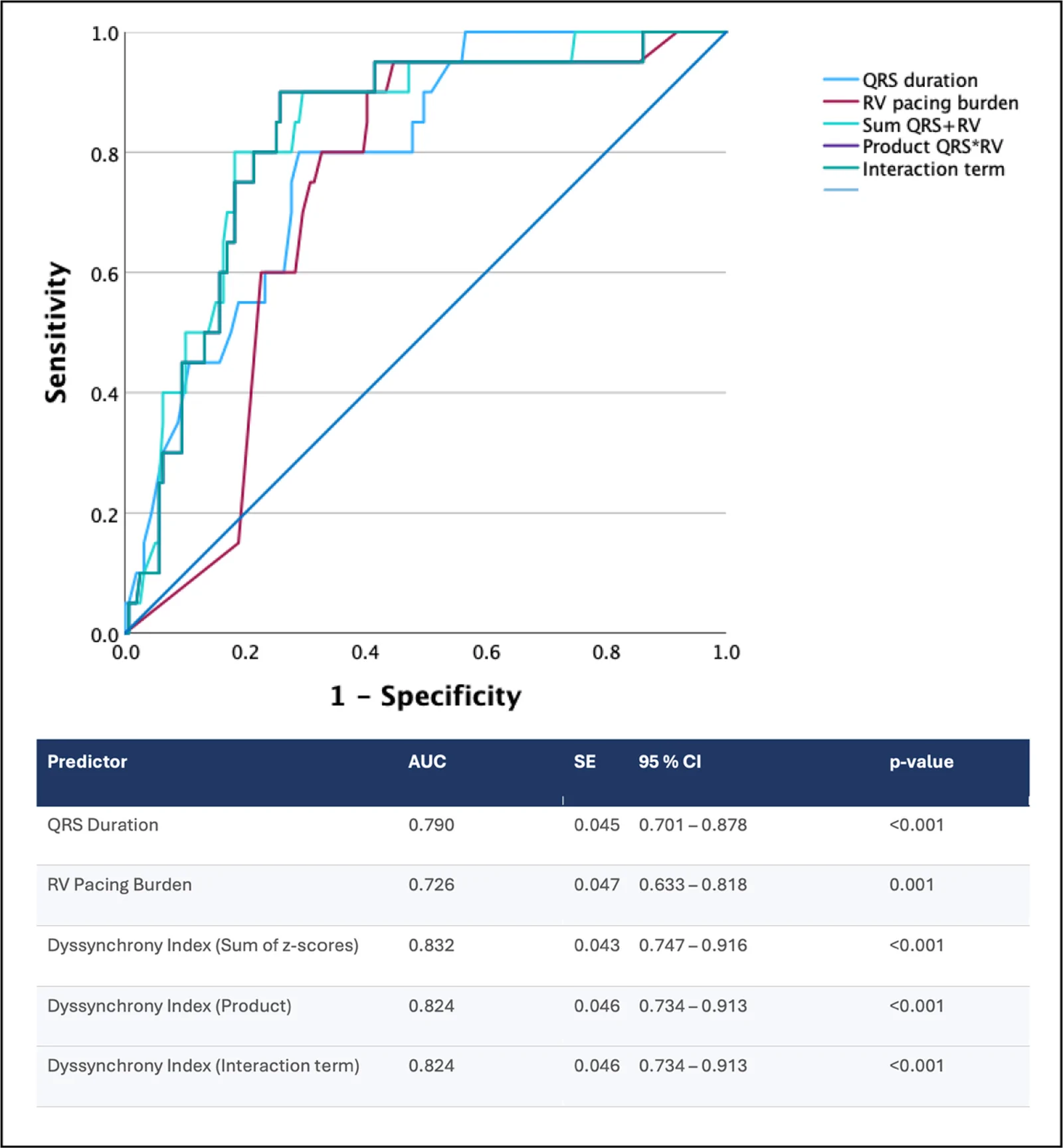
**ROC Curves for Paced QRS Duration, RV Pacing Burden, and Dyssynchrony Burden Indices for Two-Year All-Cause Mortality.** Figure shows receiver operator characteristic (ROC) curves for QRS duration[ms], right ventricular (RV) apical pacing burden [%], standardized z-score sum of QRS duration plus RV pacing burden, product of both, and the interaction term with the area under the curve (AUC), standard error (SE), p-value, and 95% confidence interval for each predictor.

### 3.4 ROC analysis: individual and composite predictors

ROC curve analysis demonstrated that paced QRS duration (AUC 0.790, 95% CI 0.701–0.878; optimal cut-off 159 ms, 80% sensitivity, 71%specificity) and RV pacing burden alone (AUC 0.726, 95% CI 0.633–0.818; optimal cut-off 43%, 95% sensitivity, 52% specificity) each provided fair discriminatory value for two-year mortality prediction. The dyssynchrony burden index (z -score sum) achieved the highest AUC at 0.832 (95% CI 0.747–0.916; Fig. 2). The product and interaction term yielded comparable, marginally lower AUCs of 0.824 each. Formal AUC comparison (Hanley-McNeil) confirmed that the dyssynchrony index significantly outperformed RV pacing burden alone (ΔAUC 0.106, Z = 2.04, *p* = 0.042), but not QRS duration alone (ΔAUC 0.042, Z = 0.82, *p* = 0.41; Table 3).

Among echocardiographic parameters, TAPSE (AUC 0.817, 95% CI 0.748–0.887) was a good and LV-EF (AUC 0.770, 95% CI 0.680–0.860) an acceptable predictor of mortality.

### 3.5 Kaplan-meier survival analysis

Patients with a dyssynchrony burden index above the 75th percentile (*n* = 45) demonstrated markedly worse two-year survival compared to those below this threshold (*n* = 135). The high-burden group experienced 16 events, versus only 4 events in the low-burden group (log-rank *p* < 0.001; risk ratio 4.267, 95% CI 2.889–6.301), Fig. 3.

**Fig. 3:**
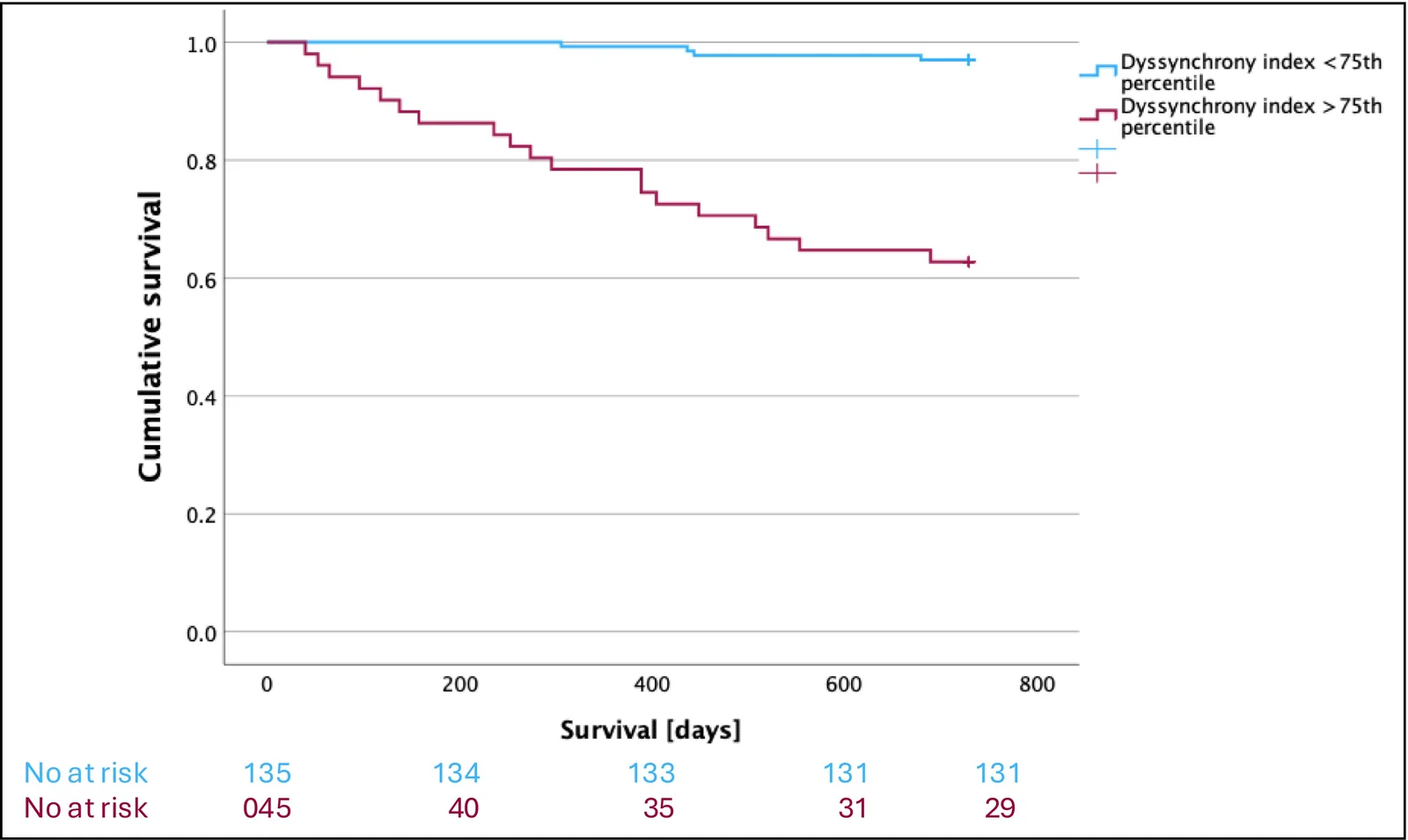
**Kaplan-Meier Survival Curves Stratified by Dyssynchrony Burden Index.** Patients were dichotomised at the 75th percentile of the dyssynchrony index: Low dyssynchrony index n = 135 (4 events), high dyssynchrony index n = 45 (16 events). Log-rank p < 0.001.

### 3.6 Multivariable cox regression

In Model A (*n* = 180), multivariable Cox proportional hazards regression identified three independent predictors of all-cause mortality: the dyssynchrony burden index (HR 3.886 per tertile increment, 95% CI 1.999–7.553, *p* < 0.001), TAPSE (HR 0.852 per mm increment, 95% CI 0.734–0.990, *p* = 0.036), and LV-EF (HR 0.920 per % increment, 95% CI 0.863–0.980, *p* = 0.010). Age and sex did not reach statistical significance in this model. In Model B (*n* = 124), substituting TAPSE with RV free-wall strain, both the dyssynchrony index (HR 3.119, 95% CI 1.228–7.923, *p* = 0.017) and RV strain (HR 0.837 per % increment, 95% CI 0.734–0.955, *p* = 0.008) remained independent predictors. Full results for both models are presented in Table 2.

**Table 2:** Multivariable Cox Regression Models for Two-Year All-Cause Mortality.

**Variable**	**HR**	**95% CI**	***p*-value**
**Model A (*n* = 180) – TAPSE as RV function parameter**			
Age [years]	1.020	0.955–1.088	0.557
Sex (male)	2.574	0.942–7.034	0.065
LV-EF [%]	0.920	0.863–0.980	0.010
Dyssynchrony index (per tertile)	3.886	1.999–7.553	<0.001
TAPSE [mm]	0.852	0.734–0.990	0.036
**Model B (*n* = 124) – RV free-wall strain as RV function parameter**			
Age [years]	1.045	0.960–1.137	0.310
Sex (male)	1.502	0.381–5.918	0.561
LV-EF [%]	0.929	0.861–1.003	0.061
Dyssynchrony index (per tertile)	3.119	1.228–7.923	0.017
RV free-wall strain [%]	0.837	0.734–0.955	0.008

Model A: *n* = 180; Model B: *n* = 124 (patients with available RV free-wall strain data). Abbreviations: HR = hazard ratio; CI = confidence interval; LV-EF = left ventricular ejection fraction; TAPSE = tricuspid annular plane systolic excursion; RV = right ventricular.

### 3.7 Combined model: dyssynchrony index and RV function

Adding RV function to the dyssynchrony index substantially improved mortality prediction. Combining the dyssynchrony index with TAPSE yielded an AUC of 0.917 (95% CI 0.858–0.976), a significant improvement over the index alone (ΔAUC 0.085, Z = 2.18, *p* = 0.030; Fig. 4A). The combination with RV free-wall strain achieved the highest discriminatory performance in this cohort (AUC 0.981, 95% CI 0.958–1.000; ΔAUC 0.149, Z = 3.62, *p* < 0.001; Fig. 4B). Formal AUC comparisons are presented in Table 3.

**Fig. 4:**
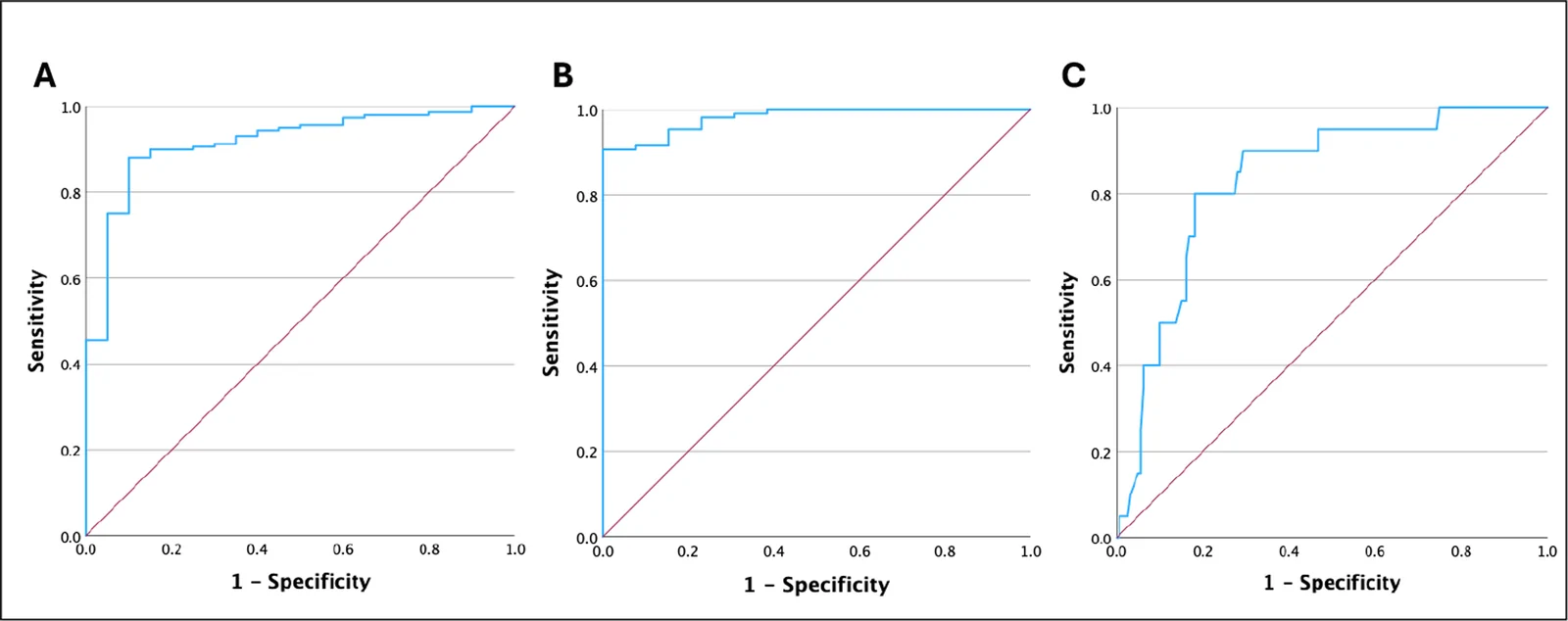
**ROC Curves for the Combined Model of Dyssynchrony Burden Index and RV Function for Two-Year All-Cause Mortality.** A) Adding TAPSE to the dyssynchrony index (C) significantly improved predictive accuracy (AUC 0.917 vs. 0.832, p = 0.030). B) The combination with RV free-wall strain achieved the highest predictive accuracy in this cohort (AUC 0.981, 95% CI 0.958–1.000; p < 0.001 vs. index alone). TAPSE = tricuspid annular plane systolic excursion. RV = right ventricular.

**Table 3:** AUC Comparison by the Hanley-McNeil Method.

**Comparison**	**ΔAUC**	**Z**	**p-value**
Dyssynchrony Index (Sum) vs. RV Pacing Burden	0.106	2.04	0.042
Dyssynchrony Index (Sum) vs. QRS Duration	0.042	0.82	0.41
Dyssynchrony Index vs. Index + TAPSE	0.085	2.18	0.030
Dyssynchrony Index vs. Index + RV Free-Wall Strain	0.149	3.62	<0.001

ΔAUC indicates the difference in area under the ROC curve relative to the dyssynchrony burden index (sum of paced QRS duration and RV pacing burden). A negative ΔAUC indicates that the comparator model had a lower AUC than the reference. AUC = area under the curve; ROC = receiver operating characteristic; RV = right ventricular; TAPSE = tricuspid annular plane systolic excursion.

### 3.8 Threshold-based point score: sensitivity analysis

Given the reliance of the primary index on cohort-specific standardisation, a simplified integer point score (range 0–2, one point each for QRS duration and RV pacing burden exceeding a threshold) was evaluated as a bedside-usable alternative. Using round clinical thresholds (QRS >150 ms, burden >40%), the point score achieved an AUC of 0.777 (95% CI 0.691–0.863); using Youden-optimal cohort thresholds (QRS >159 ms, burden >43%), the AUC was 0.796 (95% CI 0.711–0.882), Fig. 5. Both were numerically lower than the continuous z-score index (AUC 0.832, 95% CI 0.747–0.918). The clinical-threshold score differed significantly from the continuous index (ΔAUC 0.055, Z = 2.83, *p* = 0.005), whereas the Youden-threshold score did not (ΔAUC 0.036, Z = 1.86, *p* = 0.063); the two point-based versions did not differ from each other (ΔAUC 0.019, Z = 1.13, *p* = 0.261).

**Fig. 5:**
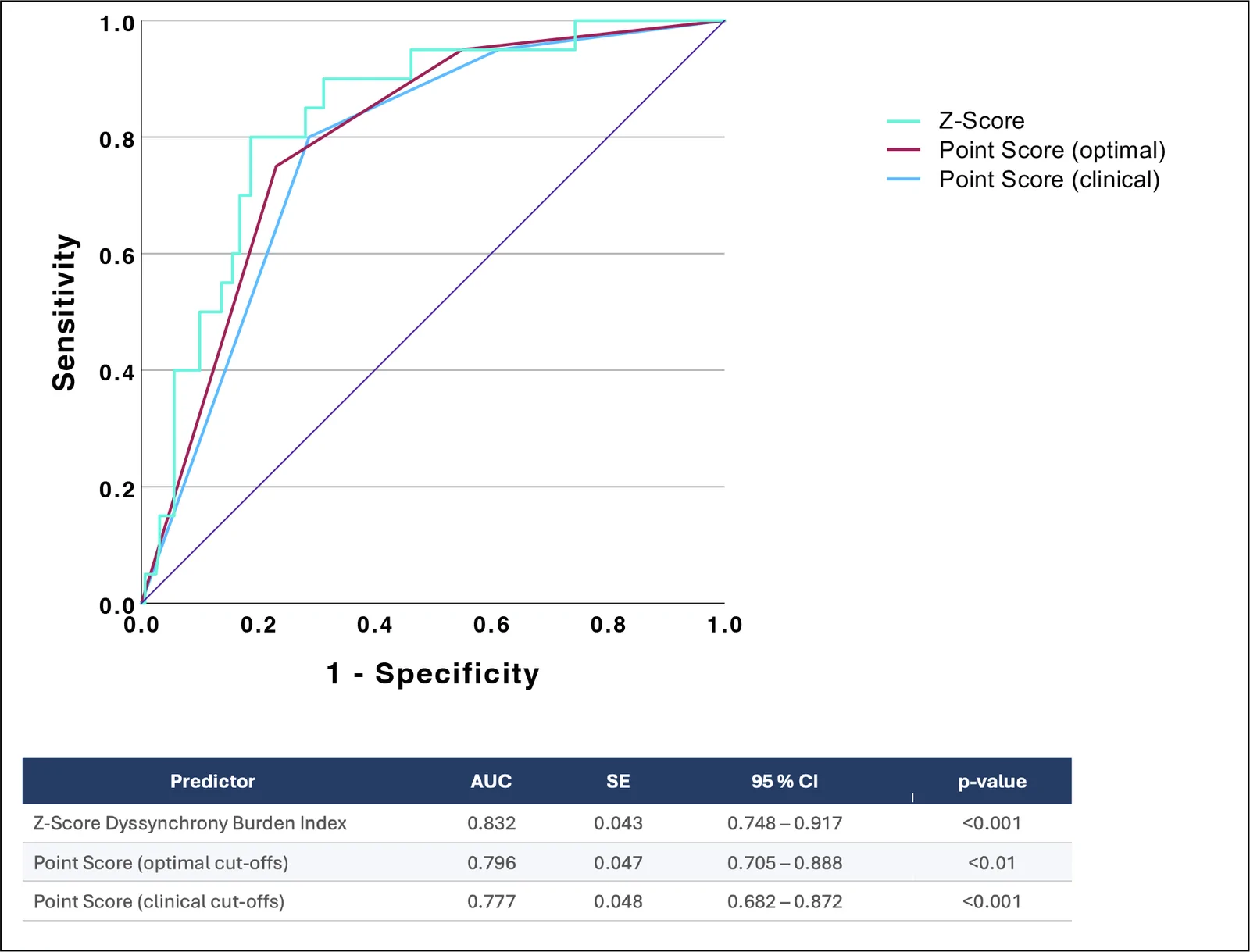
**ROC Curves for Z-Score derived Dyssynchrony Burden Index and Threshold-Based Point Scores for Two-Year All-Cause Mortality.** Figure shows receiver operator characteristic (ROC) curves for z-score sum derived Dyssynchrony Burden Index and threshold-based point scores with the area under the curve (AUC), standard error (SE), p-value, and 95% confidence interval for each predictor.

Both point scores were independently associated with two-year mortality on logistic regression (clinical-threshold score: Odds ratio 5.17 per point, 95% CI 2.13–12.54, *p* < 0.001; Youden-threshold score: Odds ratio 5.31 per point, 95% CI 2.32–12.11, *p* < 0.001). At a cut-off of ≤1 point, both scores functioned as a highly sensitive rule-out for mortality (sensitivity 95%, negative predictive value ≥98%), while a score of 2 (both criteria present) provided a more balanced rule-in threshold (specificity 71–77%, positive likelihood ratio 2.8–3.2).

Survival according to threshold-based point scores is shown in Fig. 6.

**Fig. 6:**
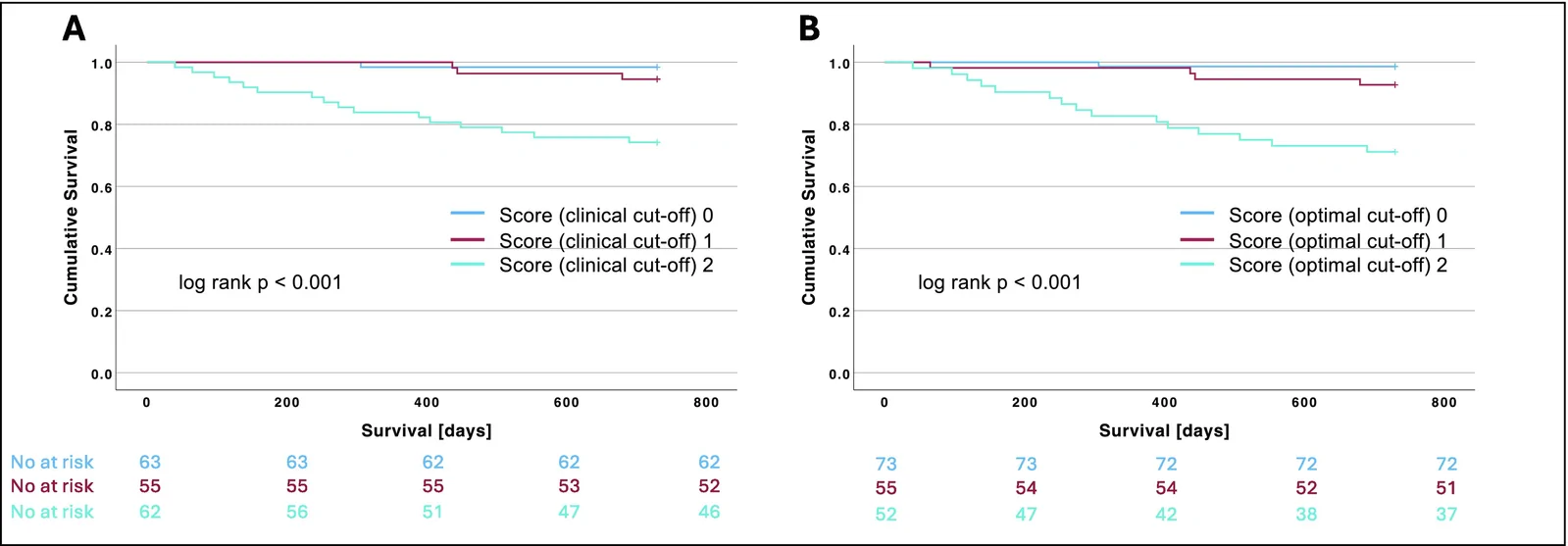
**Kaplan-Meier Survival Curves stratified by Threshold-Based Point Scores.** Patients were stratified according to threshold-based point scores (0–2) **A)** with clinical cut-off (QRS > 150 ms, right ventricular apical pacing > 40%) and **B)** with optimal (Youden Index) based cut-off (QRS > 159 ms, right ventricular apical pacing > 43%).

**Internal validation via bootstrap resampling**

Given the limited number of events relative to the number of covariates in the multivariable Cox models (Model A: 20 events for 5 covariates, 4.0 events per variable; Model B: 13 events for 5 covariates in the *n* = 124 subset with available RV strain data, 2.6 events per variable), internal validation was performed using bootstrap resampling (1000 resamples). For Model A, the apparent Harrell's C-statistic was 0.908, the optimism-corrected C-statistic was 0.895, and the bootstrap-derived shrinkage factor was 0.844, indicating modest optimism. For Model B, the apparent C-statistic was 0.960, the optimism-corrected C-statistic was 0.940, and the shrinkage factor was 0.686; in addition, 35 of 1000 bootstrap resamples (3.5%) produced unstable or near-separated coefficient estimates, providing direct, quantitative evidence of the overfitting risk associated with this smaller subgroup.

## 4 Discussion

### 4.1 Main findings

This study introduces a dyssynchrony burden index – defined as the arithmetic sum standardized paced QRS duration (ms) and RV pacing burden (%) and two simplified, bedside-calculable point-based versions preserving most of its prognostic information – as independent predictor of two-year all-cause mortality in patients with permanent RV apical pacing and preserved LV systolic function. The continuous index significantly outperformed RV pacing burden alone. Patients in the highest quartile (index >1.644) experienced markedly worse two-year survival, with a mortality rate of >30% versus ≤5% in each lower quartile. Both point-scores were independently predictive with only modestly lower discrimination. A point score ≤ 1 was highly sensitive for rule-out whereas a score of 2 reasonably specific for rule-in.

On multivariable Cox regression, the dyssynchrony index, TAPSE, and LVEF were independent predictors. RV strain independently predicted mortality when substituted for TAPSE. Combining the electrical index with either RV functional parameter substantially improved discrimination.

### 4.2 Mechanistic and theoretical interpretation

The construct of the dyssynchrony burden index rests on the mechanistic rationale that the pathological consequences of RV apical pacing are proportional not to a single electromechanical feature but to the cumulative electrical stimulus imposed on the myocardium over time. Paced QRS duration reflects the spatial extent of ventricular depolarisation per individual heartbeat, while RV pacing burden encodes the temporal density of this exposure, i.e., the proportion of cardiac cycles during which this activation pattern prevails. By summing these two dimensions, the index approximates a dose-effect relationship, capturing both the intensity and the chronicity of the dyssynchronous electrical stimulus in a single composite metric.

This mechanistic framing is supported by converging lines of experimental and clinical evidence. Animal models of chronic RV apical pacing demonstrate that myocardial fibrosis and mitochondrial dysfunction accumulate in proportion to pacing duration and are more pronounced when artificially widened QRS complexes are imposed [14], [15]. Clinically, the seminal MOST trial observation that each percentage-point increment in ventricular pacing confers a graded increase in heart failure risk is conceptually analogous to the burden dimension of the present index [4]. Similarly, the positive correlation between paced QRS width and adverse LV remodelling has been reproduced in multiple registries [10]. The dyssynchrony burden index provides an integrated operationalisation of both dimensions simultaneously.

RV dysfunction in the context of chronic RV apical pacing is mediated through at least three interacting mechanisms. First, direct mechanoenergetic uncoupling occurs because the RV myocardium is abnormally activated from the apex, reversing its normal base-to-apex contractile sequence and generating regional contraction-relaxation heterogeneity [1]. Second, ventricular interdependence propagates LV dyssynchrony-induced septal dyskinesia into the RV, impairing RV filling and systolic performance [11]. Third, pacing-induced LV dysfunction and secondary mitral regurgitation elevate left atrial pressures, with ensuing pulmonary hypertension imposing increased afterload on the RV. TAPSE and RV strain capture distinct facets of this process: TAPSE reflects predominantly longitudinal annular excursion driven by the tricuspid ring and infundibular myocardium, whereas RV strain, derived by speckle-tracking, measures the aggregate shortening of the RV free wall independent of translational motion – rendering it more sensitive to early and regional RV dysfunction [16], [17]. The superior discriminatory capacity of RV strain over TAPSE in the present study aligns with a growing body of evidence positioning strain imaging as a more sensitive RV biomarker in structurally compromised hearts [18].

### 4.3 Contextualisation within existing literature

The present findings corroborate and extend several important prior studies. Khurshid et al. demonstrated in a large cohort that PICM occurs in up to 20% of pacemaker patients and is predicted by pacing burden, QRS duration, male sex, and bundle branch block morphology [10]. The present study refines this by showing that the combination of burden and QRS duration into a single index improves discrimination beyond either alone and by shifting the primary outcome from LV remodelling to all-cause mortality. Merchant and Mittal showed that RV pacing burden above 40% is associated with significant echocardiographic deterioration, a threshold that aligns with the disproportionate mortality risk observed in the present cohort [7].

The prognostic significance of TAPSE in the paced patient population has been less systematically explored than in heart failure, tricuspid regurgitation, or pulmonary hypertension cohorts. TAPSE may provide prognostic information complementary to LV systolic function alone. The incremental value of RV strain over TAPSE resonates with recent retrospective data demonstrating that speckle-tracking RV parameters predict outcomes beyond conventional indices [18]. The fact that RV strain retained independent prognostic significance in the multivariable model suggests that strain imaging captures pathophysiological processes not fully reflected in annular excursion, such as subendocardial fibrosis and early myocyte dysfunction.

Critically, this study's restriction to patients with LVEF ≥40% distinguishes it from most PICM registries that include subjects with established LV systolic impairment. By excluding relevant pre-existing LV dysfunction, the population isolates the effect of pacing-induced dyssynchrony from pre-existing structural heart disease, rendering the mortality associations more directly attributable to the haemodynamic consequences of RV apical pacing per se. The 11.1% two-year mortality rate in this relatively preserved-function cohort underscores that pacing burden and associated RV dysfunction may carry clinically significant mortality risk even in the absence of overt LV failure – a finding with direct implications for clinical monitoring strategies in the growing population of pacemaker-dependent patients. However, the number of comorbidities, markedly elevated NT-proBNP levels, and rather broad intrinsic QRS complexes indicate that patients in this cohort had an overall advanced disease burden. Effects may have been therefore more pronounced and not be comparable to healthier individuals.

### 4.4 Clinical applicability: a simplified point-based score

A cohort-relative z-score sum, while achieving the best discrimination in this dataset, cannot be reconstructed by a clinician at the bedside and its numerical value is not portable across cohorts with different underlying QRS duration and pacing burden distributions. We therefore evaluated a simple, integer point-based score derived from fixed clinical thresholds. This point score sacrificed only a modest, and in the case of the Youden-derived thresholds statistically non-significant, amount of discrimination relative to the continuous index, while remaining strongly and independently associated with mortality and offering directly interpretable rule-out (score ≥ 1) and rule-in (score = 2) performance. We propose that the continuous z-score sum remains valuable as the best-discriminating research index in this cohort, while the threshold-based point score offers a pragmatic, externally calculable tool that addresses the concern that a cohort-derived standardisation cannot be applied prospectively to an individual patient without access to the derivation cohort's mean and standard deviation.

### 4.5 AF, fusion beats, and medical therapy

AF was present in 53.3% of the cohort (42 paroxysmal, 20 persistent, 34 permanent) and did not differ significantly between survivors and non-survivors. AF subtype may influence intrinsic atrioventricular conduction, pacing mode selection, and RV pacing burden; conversely, catheter ablation restoring sinus rhythm could reduce pacing burden and thereby modify the dyssynchrony index independently of the underlying disease process. History of AF catheter ablation was not systematically available in this dataset and represents an unmeasured potential confounder that should be captured in future validation cohorts. Similarly, RV pacing burden was derived directly from device-reported percentages, which cannot distinguish genuine ventricular capture from fusion or pseudofusion beats in patients with intermittent intrinsic conduction; paced QRS duration, by contrast, was measured only from clearly fully-paced complexes, so this potential source of misclassification is asymmetric between the two components of the index and may attenuate, rather than inflate, the observed associations. Baseline guideline-directed medical therapy was common in this cohort. Given the limited number of events, these therapies were reported descriptively rather than entered into the multivariable models, and residual confounding by differential medical therapy cannot be excluded.

### 4.6 Study limitations

Several limitations of the present study warrant consideration. The retrospective single-centre design introduces susceptibility to selection bias, unmeasured confounders, and restricted generalisability. Referral patterns, institutional echocardiographic protocols, and device programming practices may not be representative of other centres, and prospective multicentre validation is required before the dyssynchrony burden index can be adopted as a clinical decision support tool, including the newly developed simplified point score.

The relatively small sample size (*n* = 180) with only 20 mortality events limits statistical power, increases the risk of overfitting in multivariable models, and precludes meaningful subgroup analyses by pacing indication, programming mode, or comorbidity burden. The observed hazard ratios and AUC estimates carry wide confidence intervals and should be interpreted cautiously pending replication in larger, independent cohorts.

Bootstrap internal validation quantified this concern directly. Model A (5 covariates, 4.0 events per variable) showed modest optimism. Model B (5 covariates, 2.6 events per variable in the *n* = 124 subset with RV strain data) showed more pronounced overfitting. Model Bs hazard ratios and its apparently very high AUC when combined with RV strain should therefore be regarded as substantially more susceptible to optimism than Model A. Patients with available strain data may differ systematically from those without potentially inflating the apparent discriminatory performance of the strain-containing model.

The dyssynchrony burden index is an arithmetic sum of two variables with formally incommensurable units (milliseconds and percent), and its superiority over paced QRS duration alone did not reach statistical significance. The index is best understood as a clinically pragmatic synthesis rather than a physiologically derived composite, and alternative weighting schemes – such as normalising each component to its standard deviation or deriving weights from a derivation dataset – should be explored in future work.

Pacemaker indication (e.g., complete AV block versus sick sinus syndrome), mode programming evolution over the follow-up period, and the use of algorithms to minimise unnecessary RV pacing were not systematically reported. These factors may confound pacing burden estimates and introduce heterogeneity in the exposure-outcome relationship. Furthermore, all-cause mortality does not distinguish cardiac from non-cardiac causes of death, and competing risks analyses were not performed.

Only all-cause mortality was recorded in this dataset; cause-specific (cardiac versus non-cardiac) mortality data were not available. We consider all-cause mortality a robust and clinically relevant endpoint in this elderly, comorbid population, in whom adjudication of cause of death is itself often uncertain, but we acknowledge that this precludes distinguishing arrhythmic, heart-failure-related, and non-cardiac deaths. Additionally, follow-up was calculated from the date of the index device interrogation and echocardiographic assessment rather than from pacemaker implantation; this design reflects routine clinical practice, in which risk assessment typically occurs at a follow-up visit rather than immediately post-implant, but means the study cannot characterise the acute effects of a new pacemaker implant on the study outcomes.

A median of ∼3 years elapsed between pacemaker implantation and the index assessment defining time zero in this study. Because patients were required to survive to this index assessment to be included, the cohort is a *prevalent*, not an *inception*, sample with respect to the implant date. Patients who died in the interim, including from causes plausibly related to the pacing exposure itself, were necessarily excluded, since they never reached the baseline assessment from which follow-up was measured. This constitutes a form of left truncation (survival bias): the analysis by design excludes the patients at highest early risk from RV apical pacing, retaining only those robust enough to survive roughly three years of pacing exposure before entering the study. As a consequence, the associations reported here likely represent a conservative, lower-bound estimate of the true relationship between pacing-induced dyssynchrony and mortality. This is a structural limitation of any retrospective cohort anchored to a device-interrogation or echocardiographic assessment rather than to implantation itself, and it cannot be corrected post hoc.

Finally, the two-year follow-up period, while sufficient to detect between-group survival differences in this high-risk cohort, may underestimate long-term prognostic separation. Longer follow-up is needed to determine whether the dyssynchrony burden index retains predictive power beyond two years and to capture late events in lower-risk strata, especially since pacemaker therapy may be relevant for decades in single patients. External validation in independent prospective cohorts is mandatory before clinical translation.

### 4.7 Future directions and clinical implications

If validated prospectively, the dyssynchrony burden index – calculable from two parameters routinely available at any device follow-up visit without additional cost or infrastructure – could serve as a practical risk stratification tool to identify patients who may benefit from prophylactic intervention prior to overt PICM development. Candidate interventions could include upgrade to cardiac resynchronisation therapy (CRT), transition to His-bundle pacing, or left bundle branch area pacing (LBBAP), and optimisation of AV and VV delays to minimise paced QRS width. Importantly, physiological pacing strategies such as His-bundle pacing and LBBAP restore near-normal ventricular activation and substantially narrow the paced QRS complex, directly attenuating the per-beat dyssynchrony component of the proposed index [19]. Randomised trials comparing physiological versus RV apical pacing with prospective tracking of the dyssynchrony burden index as both a stratification variable and a serially measured exposure endpoint would be of scientific and clinical value.

The high combined discriminatory performance of the dyssynchrony index together with RV function suggests a potential model for a structured follow-up protocol: patients exceeding the 75th percentile dyssynchrony index threshold could be triaged to advanced echocardiographic assessment including speckle-tracking RV strain, with the combined risk estimate. However, prospective implementation studies are needed to evaluate the feasibility, cost-effectiveness, and clinical impact of such a protocol.

From a translational perspective, future studies should clarify the biological determinants of the observed partial independence between dyssynchrony burden and RV dysfunction. Cardiac magnetic resonance imaging with late gadolinium enhancement and T1 mapping could quantify myocardial fibrosis in relation to the dyssynchrony index, potentially identifying a structural substrate that precedes functional deterioration and is amenable to early intervention [20].

Biomarker studies incorporating GDF-15, soluble ST2, and galectin-3 could complement the proposed multiparameter model, providing mechanistic insight into the dyssynchrony-remodelling-mortality pathway and potentially enabling blood-based risk stratification as an adjunct to imaging [21].

## 5 Conclusions

The dyssynchrony burden index – a composite of paced QRS duration and RV pacing burden – is an independent predictor of mortality in patients with RV apical pacing and preserved LV systolic function. It reflects cumulative electrical exposure and provides independent and additive prognostic information. The combination of the dyssynchrony burden index with echocardiographic RV functional parameters substantially improves mortality prediction, supporting a multiparameter approach to risk stratification in this population. A simplified, bedside-calculable point-based version of the index (based on QRS duration and RV pacing burden thresholds), evaluated as a sensitivity analysis, retained strong, independent prognostic value with only a modest reduction in discrimination.

## CRediT authorship contribution statement

**Marwin Bannehr:** Writing – original draft, Visualization, Methodology, Investigation, Formal analysis, Data curation, Conceptualization. **Laura David:** Validation, Software, Investigation, Data curation. **Christoph Edlinger:** Writing – review & editing, Methodology, Conceptualization. **Christian Georgi:** Writing – review & editing, Methodology. **Paulina Karlsson:** Data curation. **Tanja Kücken:** Data curation. **Martin Seifert:** Writing – review & editing, Supervision, Project administration, Methodology. **Anja Haase-Fielitz:** Writing – review & editing, Validation, Supervision, Project administration, Methodology, Investigation, Formal analysis, Conceptualization. **Christian Butter:** Writing – review & editing, Supervision, Project administration.

## Declaration of competing interest

The authors declare the following financial interests/personal relationships which may be considered as potential competing interests: MB (corresponding author) received educational grants from Boston Scientific and Medtronic, CG (co-author) from Boston Scientific and Biotronik. PK (co-author), and MS (co-author) received educational grants from Boston Scientific. If there are other authors, they declare that they have no known competing financial interests or personal relationships that could have appeared to influence the work reported in this paper.
